# Exploring the Accuracy and Consistency of a School Readiness Assessment Tool for Preschoolers: Reliability, Validity and Measurement Invariance Analysis

**DOI:** 10.3390/jintelligence11100189

**Published:** 2023-09-28

**Authors:** Krisztián Józsa, Tun Zaw Oo, Diana Borbélyová, Gabriella Zentai

**Affiliations:** 1 Department of Primary and Pre-School Education, J. Selye University, 94501 Komárno, Slovakia; borbelyovad@ujs.sk; 2 Institute of Education, University of Szeged, 6722 Szeged, Hungary; 3 Institute of Education, Hungarian University of Agriculture and Life Sciences, 7400 Kaposvár, Hungary; oo.tun.zaw@uni-mate.hu (T.Z.O.); zentai.gabriella@uni-mate.hu (G.Z.)

**Keywords:** school readiness, DIFER, reliability, validity, measurement invariance, assessment

## Abstract

This study focuses on examining the psychometric properties of the DIFER test, a widely used assessment tool for measuring school readiness. DIFER, which stands for Diagnostic Assessment Systems for Development, has gained prominence in Hungary and some European countries as an effective means of evaluating children’s readiness for school. By investigating the reliability and validity of the DIFER test, this study aims to enhance the understanding of the suitability of the DIFER test for cross-cultural and longitudinal studies in assessing school readiness. Conducted as a survey study, the research involved 3050 Hungarian students from Slovakia and Hungary. Employing Rasch analysis and multi-group confirmatory factor analysis (MG-CFA) aid in verifying the precision of the DIFER test as a valuable assessment instrument for determining school readiness. The results revealed a strong alignment between the difficulty level of the test and students’ actual abilities, demonstrating its reliability and validity. Importantly, the analysis found measurement invariance across various factors, including country, gender, and age. This indicates the consistent performance of the DIFER test in assessing school readiness across diverse groups. However, mean differences in latent abilities were observed among different age groups, indicating that older students exhibited notably higher proficiency in pre-mathematical skills compared to their younger counterparts. The findings offer valuable insights to educators, providing a reliable tool for assessing school readiness and identifying areas for improvement.

## 1. Introduction

The transition from the early stages of exploration and discovery to the structured expectations of formal education signifies a critical juncture in a child’s educational journey. It is during this pivotal period that the concept of school readiness takes center stage, acting as a vital determinant of a child’s future academic success (Macy et al. 2022). School readiness encompasses a comprehensive set of foundational abilities that encompass diverse domains of early learning, including cognitive skills, receptive and expressive language proficiency, executive functions, and social–emotional and behavioral competencies (Amukune et al. 2022a; Józsa et al. 2022a; Russo et al. 2019).

Evaluating school readiness assumes paramount significance, as it provides crucial insights into a child’s preparedness for the educational journey that lies ahead. Children who enter school without the necessary skills and competencies required for school readiness often experience challenges in their developmental trajectory, potentially hindering their academic progress throughout their elementary school years (Russo et al. 2019). To this end, a variety of assessment approaches have been developed to gauge children’s readiness for school, including the game-based assessment (GBA) by Amukune et al. (2022a); the Brief Early Skills and Support Index (BESSI) by Fink et al. (2019); and the Diagnostic Assessment Systems for Development (DIFER) introduced by Nagy et al. (2004a) and explored by Józsa et al. (2022b).

In the era of globalization, researchers have been afforded numerous opportunities to conduct cross-cultural studies (Anthony et al. 2022; De Los Reyes et al. 2022; Torregrosa Díez et al. 2022) and longitudinal investigations (Brock et al. 2018; Opozda-Suder et al. 2021; Samuels et al. 2016) across various educational domains. However, for such studies to yield meaningful and comparable results, it is imperative that the measurement instruments used possess measurement invariance. (Diotaiuti et al. 2022). By establishing measurement invariance, researchers gain confidence in comparing and interpreting analytical outcomes, such as latent means, across distant groups and different timeframes (Gygi et al. 2016).

Although the concept of measurement invariance has garnered considerable attention in psychological research (Bravo et al. 2021; Calchei et al. 2023; Lau et al. 2022; Teo et al. 2022; Zewude and Hercz 2022), there remains a significant research gap concerning the confirmation of psychometric properties of school readiness assessment. Consequently, the present study endeavors to address this gap by examining the measurement invariance of the DIFER test, which assesses the school readiness of young children. Through an in-depth exploration of the psychometric properties of this assessment, we aim to contribute to the body of knowledge surrounding school readiness assessment in the context of educational studies.

## 2. Literature Review

### 2.1. Children’s School Readiness and Assessment

Various approaches exist for defining or conceptualizing the essence of children’s school readiness. For example, school readiness is defined as the capacity of children to regulate emotions for appropriate social responding, as well as the ability to regulate attention and utilize selective strategies during cognitive tasks, with self-regulatory skills forming the foundation for the behaviors and attributes associated with successful school adjustment (Blair 2002; Curby et al. 2018; Denham 2006; Duncan et al. 2007; Józsa et al. 2022a; Miller and Goldsmith 2017). It also refers the acquisition of a range of skills typically anticipated upon starting school, enabling children to thrive in their social and academic growth (Bender et al. 2011; Macy et al. 2022). This multifaceted and comprehensive notion encompasses various aspects, such as physical, social, emotional, and cognitive skills and competencies. Mukkiri et al. (2022) clearly defined it as basic skills that children need to possess at school entry in order to adapt successfully to the school environment and to learn and achieve at a satisfying level. Regarding the school readiness assessment, the DIFER (the Diagnostic System for Assessing Development) school readiness test is quite popular in Hungary and some countries in Europe (Józsa et al. 2022a). The DIFER test aims to assess the progress of fundamental abilities in children aged 4–8 and to delineate the benchmarks for enhancing their acquisition (Nagy et al. 2004a):

- Fine-tuned co-ordination between writing and motion, a prerequisite for writing instruction (fine motor skills);
- Effective speech perception and auditory skills, a fundamental requirement for successful reading instruction (phoneme perception skills);
- Foundational vocabulary knowledge, essential for proficient verbal communication (reading comprehension);
- Fundamental arithmetic capabilities (pre-mathematics skills);
- Deduction based on experiential learning (deductive reasoning skills);
- Comprehension of relationships based on experimental learning, both pivotal for cognitive advancement (relational reasoning skills);
- Cultivation of social aptitudes, pivotal for school life and personality development (social skills).

Fine motor skills refer to the abilities to adeptly hold, grip, and control diverse objects. The progression of fine motor skills involves the synchronization of small muscles, particularly those within the hands and fingers (Fischer et al. 2022). Phoneme perception is quite important for the reading acquisition of preschool and young elementary school children. Their phoneme perception depends on their awareness of the segmental nature of spoken language and the ability to manipulate its constituent parts (Conant et al. 2014). Teaching school-age children the skill of reading comprehension is pivotal, as it acts as a method of transferring knowledge that gains greater significance as they progress through their academic years and into the future. Reading comprehension is an interactive process consisting of two main aspects: the ability to directly understand the text and the ability to draw conclusions (including two types of conclusions—cohesive conclusions and information-based conclusions) (Spätgens and Schoonen 2019). The pre-mathematics skills in DIFER are a combination of five different sub-skills of children such as counting-up, counting-down, manipulative counting, object counting, and number reading (Nagy et al. 2004a). Reasoning skills are quite important for young children’s academic education and future lives. Their deductive reasoning involves using known principles to establish the placement of a new object or entity within a sequence; it involves drawing a conclusion based on facts that are already known as true. And for relational reasoning, this refers to employing known relationships to deduce connections between new entities; it involves utilizing an understanding of equivalent patterns or relational comparisons to make sense of a novel pattern (Guerin et al. 2021). Finally, with regard to social skills, this encompasses the capacity to form successful and favorable interactions with peers, which are linked to a smoother transition into formal school environments and sustained academic success throughout their educational journal (Valiente et al. 2021; Ziv 2013). Hence, it is evident that the readiness of children for school plays a crucial role in their academic/school achievement, highlighting the necessity to prioritize the evaluation of school readiness to ensure accurate assessment.

Various educators worldwide employ diverse assessment methods for assessing children’s school readiness based on different knowledge and competence domains. Macy et al. (2022) utilized two recently developed measures known as AEPS-3 Ready-Set and Ready-Set Family Assessment of Children’s Skills (FACS). Ready-Set is a tool designed to evaluate children’s readiness for kindergarten, collecting information from teachers or professionals regarding their skills in essential developmental areas such as adaptive, cognitive, fine motor, gross motor, social emotional, social communication, literacy, and math. FACS serves as a companion measure, enabling parents to assess and report their child’s abilities across the same developmental areas covered by Ready-Set (Macy et al. 2022). The findings indicated that teachers perceived Ready-Set as a user-friendly resource that supplied pertinent information about children’s readiness skills. In another study, the Jamaica school readiness assessment (JSRA) test was employed (The Jamaica Education Transformation Commission 2021). JSRA comprises three components: the Eleven-Question Screen (EQS), which is an adapted version of a ten-question screening; the child behavior rating scale; and the early learning scales. It assesses developmental aspects, behavior, early literacy skills, early numeracy skills, and approaches to learning. The results showed that additional measures need to be taken to enhance and address data gaps, ensuring the validity and reliability of the data. Another study (De Almeida Maia et al. 2022) employed the Bracken School Readiness Assessment (BSRA) to evaluate six fundamental concepts through a set of 88 questions divided into six domains: colors, letters, numbers/counting, sizes, comparisons, and shapes. Researchers found clear indications of multidimensionality, showing 10 items (out of 88 items) with low reliability. Additionally, Fink et al. (2019) conducted a study that investigated the connection between social success upon entering school and teachers’ evaluations of school readiness using the Brief Early Skills and Support Index (BESSI), while also accounting for language ability. The result highlights the significance of cognitive and socioemotional abilities, as well as family support, in terms of a child’s preparedness for school and their social achievements during the transition to formal education. Chinese teachers’ perceptions were also collected for their children’s school readiness in one study (An et al. 2018). The study used the Chinese Teachers’ Judgments of Children’s Behavior Survey which has 32 questions in total. The survey questionnaire has five main parts such as questions about entering the first grade, questions about school information, questions about teacher information, questions about teacher preparation, and questions about classroom information. The results indicated that the students were not ready for school, experiencing challenges in both academic and social–emotional abilities. Moreover, in one study, the aim of the assessment was to compare the school readiness and motor abilities of typically developing first-grade students with those of disadvantaged children. Lepes et al. (2016) assessed children’s skills such as writing–motion, speech–hearing, relational vocabulary, basic calculation, socializing, deduction, and comprehension of relationships. The study found the importance of socializing and motor skills of children in their school readiness even though there is a lack of results about the reliability and validity of the instruments.

While the majority of previous studies examining the assessment of school readiness have primarily focused on the cognitive aspect, and social and motor skills, recent investigations have revealed additional crucial factors that contribute to the transition from preschool to kindergarten. These factors include motivation, executive function, and emotion regulation (Amukune et al. 2022b; Berhenke et al. 2011; Blasco et al. 2023; Józsa et al. 2017; McWayne et al. 2012). Moreover, UNICEF has generally proposed a school readiness model that encompasses three key components: school-related information, child-related information, and family/community-related information (Nair et al. 2023). To sum up, various researchers have employed diverse domains when assessing the school readiness of different student groups, with cognitive aspects, social skills, and motor skills being commonly included. It is crucial to acknowledge that these instruments need to undergo psychometric evaluation to ensure their suitability for different participants and varying timeframes (Liu et al. 2020). Additionally, emphasis should be placed on incorporating assessment theories during the development of psychological scales (Polat et al. 2022).

### 2.2. Developmental Change by Age

Understanding the trajectory of developmental change across different age groups is crucial for comprehending the nuances of cognitive and socio-emotional development. As children progress through their early years, marked shifts in cognitive abilities, emotional regulation, and social interactions occur. These developmental changes are often attributed to the interplay of genetic predispositions, environmental influences, and maturation processes (Blair and Raver 2015). Demetriou et al. (2020) emphasize the need to explore these age-related transformations, highlighting the significance of investigating how empirical factor structure evolve across different age groups. According to the age span of four years, there is a change in students’ mental process and personality (Demetriou et al. 2023). Assessing school readiness across age groups demands understanding key cognitive factors, where general cognitive ability (g) plays a crucial role. The underlying ‘g’ factor showed a significant heritability of 86%, primarily contributing to genetic influences across distinct cognitive domains and fundamental cognitive assessment (Panizzon et al. 2014). Furthermore, Neumann et al. (2021) mention that cognitive abilities evolve swiftly in the initial stages of childhood due to the maturation of the brain and the influences of the surrounding environment. As a result, it is essential to take into account age-related aspects when evaluating their developmental progress. This endeavor becomes especially pertinent in the context of assessing school readiness, as the transition to formal education coincides with a pivotal phase in a child’s development. By capturing and analyzing these developmental shifts, researchers can gain insights into the distinct cognitive, emotional, and behavioral features that characterize each group, thereby advancing our understanding of the intricate process of children’s development.

### 2.3. Theoretical Perspectives to Assessments

There are some measurement theories which can supply primary methods used in the psychological scale development. Test theories are frameworks used in psychometrics to study the properties of psychological tests and measure various aspects of human behavior (Dean et al. 2021). Three popular test theories are the classical test theory (CTT), item response theory (IRT), and structural equation modeling (SEM).

CTT is the oldest measurement theory that assumes a person’s test score is the sum of their true score (actual ability) and measurement error (Siregar and Panjaitan 2022). It analyzes the reliability, validity, and sources of measurement error, with the true score representing the individual’s actual ability and the measurement error reflecting the variability in observed scores unrelated to the true score (Haw et al. 2022). However, CTT does not account for item difficulty or variability in individual differences in ability levels (Ayanwale et al. 2022) and MI testing (Siregar and Panjaitan 2022). IRT is a modern approach to psychometric measurement that models the relationship between a person’s ability level and their responses to test items (Polat et al. 2022). IRT assumes that items have varying degrees of difficulty and discrimination, allowing the estimation of individuals’ abilities based on their responses (Liu et al. 2022). IRT is useful for analyzing differential item functioning (DIF) and detecting item bias. This DIF analysis can also be applied as one type of measurement invariance (MI) testing in some studies (Åström et al. 2022; Visser et al. 2017; Zhong et al. 2023). SEM is a statistical technique used to model complex relationships between variables. SEM is widely used in various fields, including psychology, sociology, marketing, and economics, to test and refine theories, estimate parameters, and generate predictions. Many researchers employed SEM to investigate MI across different groups, such as gender or cultural groups, to ensure that a test is measuring the same construct in all groups (AL-Dossary 2021; Anthony et al. 2022; Byrne 2016).

In the context of the DIFER test which is designed as a nationally used Hungarian school readiness test (Nagy et al. 2004a), perspectives of these three theories (CTT, IRT, and SEM) are considered to analyze the test’s properties and examine measurement invariance across different groups. CTT suggests to focus on assessing the reliability and validity of the test scores and identify sources of measurement error. And IRT is beneficial for analyzing the relationship between individuals’ abilities and their responses to test items, and identifying any items that may be biased against certain groups. Finally, SEM is appropriate for examining measurement invariance (MI) across different groups to ensure that the test is measuring the same construct in all groups.

### 2.4. Measurement Invariance (MI) and Its Assessing Methods

Measurement invariance testing can decide if the test-items can give the same challenges to test-takers of different groups or contexts (Chiu et al. 2015). MI also focuses on whether the construct of the instrument is psychometrically equal across different groups. Otherwise, measurement bias or variance shows that test-takers with the same ability or latent construct can obtain different scores depending on the group they are part of (Sočan and Kocjan 2022). Therefore, it is wise to take care with regard to the value of MI testing in psychological research. The Multi-group Confirmatory Factor Analysis (MG-CFA) is an extension on the strength of confirmatory factor analysis (CFA), providing a more comprehensive test of MI by examining multiple aspects of the construct, such as configural, metric, scalar, and residual variances (Gygi et al. 2016; Zewude and Hercz 2022).

#### 2.4.1. Configural Invariance

Configural invariance refers to the property of a measurement model that shows that the same underlying factor structure is present across different groups or time points (Fischer and Karl 2019). To test the configural invariance, we can conduct separate CFAs for each group or time points and compare the resulting models (Tsaousis and Alghamdi 2022). The fit of each model is evaluated by using goodness-of-fit indices, such as the ratio of Chi-square by degrees of freedom, the comparative fit index (CFI), the Tucker–Lewis index (TLI), the root mean square error of approximation (RMSEA), and standardized root mean square residual (SRMR) (Li et al. 2019). If the factor structure is the same across groups or time points, the model should fit the data well, indicating configural invariance (Gygi et al. 2016; Kim et al. 2022).

#### 2.4.2. Metric Invariance

Metric invariance refers to the degree to which the factor loadings are equivalent across groups or settings. If the instrument has metric invariance, the participants across groups ascribe the same meaning to the latent construct under study (Tsaousis and Alghamdi 2022). When the metric is invariant, it means that the relationship between items and the latent construct being measured is the same across groups or contexts, and that the items are measuring the same underlying construct (De Beer et al. 2022). This is important because, if the metric is not invariant, differences in scores between groups or contexts may be due to differences in the measurement properties of the instrument rather than true differences in the construct being measured (Bravo et al. 2021).

#### 2.4.3. Scalar Invariance

Investigating whether mean-responses (intercepts) for corresponding items are similar or not across groups or contexts gives us the scalar invariance. In other words, scalar invariance means that the same score on the instrument should represent the same level of the underlying construct across groups or contexts (Throuvala et al. 2021). If the item intercepts, factor loadings, and item residuals are all equal across groups, it is noted as the full scalar invariance, “when the parameters—at least two indicators per construct (i.e., loadings for partial metric invariance and loadings plus intercepts for partial scalar invariance) are equal across groups” (Cieciuch and Davidov 2015, p. 85). In psychological research, partial scalar invariance was sufficient for making the meaningful comparisons across groups or contexts (Chen 2007; Chen et al. 2018).

#### 2.4.4. Residual Invariance

Residual invariance is known as strict invariance, and refers to the similar item residuals from the metric and scalar invariant levels (Putnick and Bornstein 2016). In other words, it refers to the degree to which the residuals (i.e., the difference between the predicted values and the observed values) of a statistical model are the same across different subgroups of the data (Zewude and Hercz 2022).

### 2.5. Latent Mean Differences

If the configural invariance, factor loading invariance, and intercept invariance were established, the latent mean differences across two groups can be examined in a model in which the factor loadings and intercepts were constrained to be equal (Teo et al. 2022). Latent mean difference refers to the difference in the means of the latent variables (i.e., unobserved variables) between two or more groups in MG-CFA (Kim et al. 2022). Assessing the latent mean difference for MI typically involves a series of steps, including testing for configural invariance (i.e., the same factor structure across groups), followed by testing for metric invariance (i.e., the same factor loadings across groups), scalar invariance (i.e., the same intercepts across groups), and, finally, latent mean invariance (i.e., the same latent means across groups) (Kang and Leung 2022).

### 2.6. Background Information

In Hungary, preschool and kindergarten education is provided free of charge to all children by the government. The kindergarten period spans three years, starting at the age of 3 until the age of 6, with some flexibility in age requirements (Józsa et al. 2018). It is compulsory for children to attend kindergarten for a minimum of 4 h per day from the age of 3, and most children attend for the entire day (Nagy et al. 2018). In 2014, 97% of four-year-old children in Hungary were enrolled in kindergarten (OECD 2016; Józsa and Barrett 2018). Hungary implements social support for school attendance by offering textbooks at no cost (Langer-Buchwald 2020).

In Slovakia, compulsory national preschool education was employed, and public education was all free at all levels except for a small charge for meals (Pupala et al. 2022). The government established the first national curriculum in 1964 for ECEC services for 3- to 6-year-old children (Herlina 2010). Currently, up to 93% of kindergartens in Slovakia are in the public sector, and are state and local-government funded (Štatistická ročenka–materské školy 2019). In Slovakia, kindergarten attendance is full-time from eight a.m. to four p.m.; all of that time is educational and organized into segments (European Commission/EaCEa/Eurydice 2020). Children spend approximately eight hours a day at kindergarten (half-day attendance is also an option, but take-up is limited) (Pupala et al. 2022).

### 2.7. Context of the Current Study

In Hungary, various research studies have been conducted on students’ school readiness assessment, focusing on different domains or assessment contents. For instance, several decades ago, the renowned researcher Nagy (1976) conducted a nationwide survey on school readiness using the PREFER (Preventive Development Assessment System for Children) with a sample size of 10,000 participants (Józsa et al. 2022b). The findings were deemed reliable, and the PREFER test became established as a standardized national assessment (Józsa et al. 2022a; Nagy 1980). Later, beyond the 20th century, Nagy and his colleagues modified the PREFER test into the DIFER (Diagnostic System for Evaluating Development) test, involving 23,000 children aged 4–8 years. This test also gained recognition as a criterion-referenced assessment for the entire country (Nagy et al. 2004a). The DIFER test evaluates seven subskills of children’s development, including pre-math, fine motor control, phoneme perception, understanding of cause and effect, deductive reasoning, the vocabulary of relations, and social skills (Józsa et al. 2022a). Subsequently, the DIFER test was computerized and employed in the developmental assessment of children, with researchers utilizing different sub-skill assessments of the DIFER test based on their specific research contexts, as outlined in Table 1.

Table 1 displays numerous studies conducted on school readiness assessments of young Hungarian students using various test formats, including paper-based and computer/tablet-based tests. Among all the studies on school readiness, some are longitudinal studies (Józsa et al. 2022a; Molnár and Hermann 2023; Putnick and Bornstein 2016), some are cross-cultural studies (Amukune et al. 2022b; Józsa et al. 2017, 2022b), and some are simple and national survey studies (Csapó et al. 2014; Józsa and Fenyvesi 2006; Nagy 1976; Nagy et al. 2004b). The majority of studies employed the DIFER test to assess different domains/skills related to children’s school readiness. However, information on the assessment of psychometric properties of the test, particularly measurement invariance testing, was limited across the studies. One cross-cultural study (Amukune et al. 2022b) examined measurement invariance across countries (Hungary and Kenya) but utilized a different assessment tool called CHEXI instead of DIFER. Another study (Csapó et al. 2014) employed the DIFER test but primarily focused on examining the media effect through measurement invariance analyses. As a result, there is a significant research gap concerning the evaluation of the psychometric properties for the DIFER test. Thus, the present study aimed to address this research gap by investigating the following research questions:

RQ_1_: Do students’ abilities align with the ability levels of items in the DIFER test?
RQ_2_: What is the extent of the reliability and validity exhibited by the DIFER test?
RQ_3_: Are there any noteworthy variations in performance on the DIFER test based on factors such as countries, genders, and ages?

## 3. Materials and Methods

### 3.1. Participants

The study encompassed a sample of young Hungarian students aged 4–8 years residing in Slovakia and Hungary. In total, 382 schools (8 students per school) are included in our study. Therefore, there is a total of 3050 participants (after removing missing information from six participants), with 1609 students from Slovakia (52.75%) and 1441 students from Hungary (47.25%). Of these participants, 1641 were male students (53.82%), while the remaining 1409 students were females (46.18%). The sample was further divided into different age groups, with 282 students (9.24%) being 4 years old, 652 students (21.37%) being 5 years old, 832 students (27.27%) being 6 years old, 690 students (22.62%) being 7 years old, and 594 students (19.48%) being 8 years old. We have organized the participants into distinct categories based on their countries, segmented further by both gender and age groups (Table 2).

### 3.2. Instrument and Procedure

To measure Hungarian students from both Hungary and Slovakia, an assessment called DIFER (Diagnostic Assessment Systems for Development) is employed for children aged 4–8 years (Nagy et al. 2004a). This assessment test serves as a widely accepted evaluation of children’s school readiness. DIFER is designed to assist educators in fostering the development of six crucial skills necessary for school-based learning (Nagy et al. 2004b). These skills encompass (1) pre-mathematics (58 items), (2) fine motor skills (24 items), (3) phoneme perception (15 items), (4) deductive reasoning (16 items), (5) relational reasoning (24 items), and (6) social skills (20 items). In total, the DIFER test includes 157 items. These skill assessments of DIFER test were divided into two types of assessment: dichotomous test and rating test. The DIFER test battery underwent establishment via a nationally representative sample comprising over 23,000 children aged 4–8 years (Nagy et al. 2004b). The DIFER program package follows a criterion-based approach, wherein a predetermined criterion for each skill is established. When the attainment of this criterion for a specific skill is identified, the skill is progressed, leading to its optimal functioning. Moreover, the program is diagnostic in nature, as it furnishes insights into every facet of skill acquisition levels. The diagnostic map for skill development delineates the components of a skill that a child has already mastered and those that require further enhancement. Attaining a test with successful outcomes denotes the comprehensive and optimal acquisition and practice of skills, exemplified by nearly perfect results around 100%. In simpler terms, a child’s developmental stage is inferred based on the established optimal criterion for the particular skill. The tests were administered by trained MA in Education students in two face-to-face sessions, taking an average of 15–20 min per session. In addition, the study’s ethical approval was obtained by the University Ethics Committee.

#### 3.2.1. Dichotomous Test of DIFER

**Figure 1:**
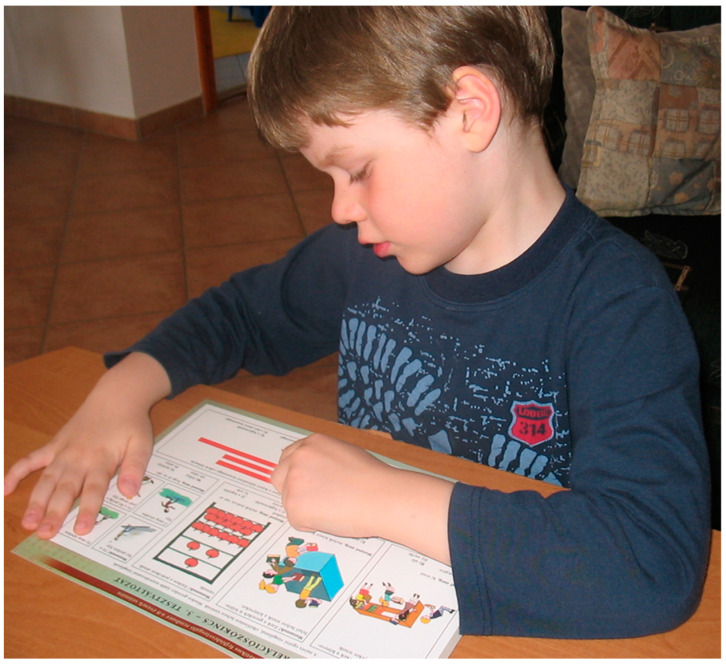
An example of dichotomous test and the test situation.

Teachers or examiners assessed students’ school readiness skills (pre-mathematics, fine motor skills, phoneme perception, deductive reasoning, and relational reasoning) using dichotomous scaled questions. An example image of the test situation is provided below (Figure 1).

#### 3.2.2. Rating Test of DIFER

**Figure 2:**
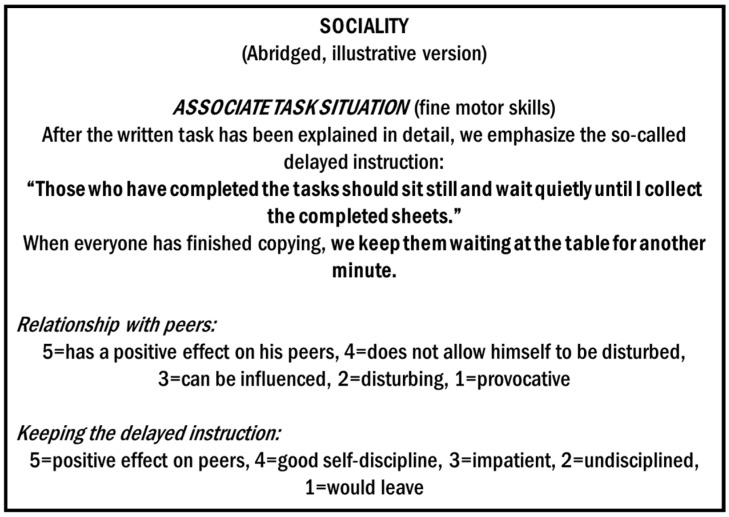
An example of rating test.

The assessment of social skills in the DIFER test involved examiners or teachers using a five-point rating scale to evaluate students’ school readiness. An illustrative image of the assessment format is presented below (Figure 2).

### 3.3. Analysis

Conquest and Winsteps software programs were utilized in this study to conduct Rasch analysis. To evaluate the quality of the DIFER test, separation values were examined, with values greater than 2 logits being considered desirable. A higher separation index indicates higher test quality, as outlined by Planinic et al. (2019). The mean square values of infit and outfit (MNSQ) were also considered, with an acceptable range typically falling between 0.5 and 1.5, although values up to 1.6 can still be regarded as acceptable. Additionally, the idea values for fit criteria were expected to be close to 1.00 logits. Furthermore, the raw residual correlation between pairs of items was evaluated, with a threshold of less than 0.3 being deemed acceptable (Boone et al. 2014). This study employed MG-CFA using SmartPLS4 and Mplus8 software packages, with additional reliability and validity measures conducted using IBM SPSS Statistics 23.0. The model fit was evaluated based on recommended fit indices; χ^2^/df < 5, RMSEA < 0.06, SRMR < 0.08, TLI > 0.90, and CFI > 0.90 (Oo et al. 2021). The invariance of the test was assessed by a change in CFI (∆CFI) of less than 0.01, a change in SRMR (∆SRMR) of less than 0.03, and a change in RMSEA (∆RMSEA) of less than 0.015, indicating the evidence of metric, scalar, and residual invariances (Bravo et al. 2021; Gygi et al. 2016; Throuvala et al. 2021).

### 3.4. Preliminary Analyses

Before conducting our main analyses, we conducted preliminary checks on the data to address missing values and assess normality. After handling any missing values in our dataset, we examined the normality of the data using skewness and kurtosis values. We found that all dimensions of the DIFER test fell within the acceptable range of −2 and +2 (Table 3), indicating that they satisfied the assumption of normality (Kline 2015).

## 4. Results

### 4.1. Addressing RQ_1_

**Figure 3:**
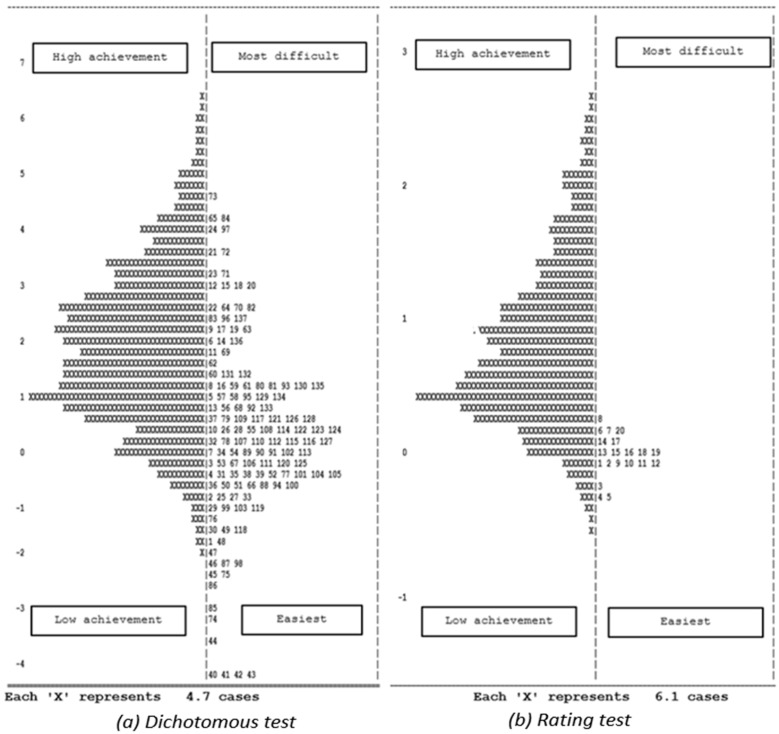
Item-person maps of DIFER.

The primary objective of this research question was to examine the item-person parameters, which would shed light on the relative difficulty or ease of specific items in the DIFER school readiness test. The DIFER assessment comprises two types of tests, namely, a dichotomous test (evaluating five domains: fine motor skills, phoneme perception skills, pre-mathematics skills, relational reasoning skills, and deductive reasoning skills) and a rating test (assessing the social skills domain). To conduct our analysis, we employed the Rasch analysis through the Conquest program, generating two models (item-person maps) for the DIFER school readiness assessment (Figure 3).

Figure 3 presents a visual depiction of the analysis outcomes. The left-hand sides of the graphs portray the students’ achievement levels or ability points, while the right-hand sides signify the difficulty levels of the test items. Notably, the graphs illustrate that students tended to exhibit higher achievement on items of moderate difficulty, indicating their proficiency in tackling items that neither posed excessive difficulty nor were excessively easy. However, it is worth highlighting that within the dichotomous test, five items (numbered 40, 41, 42, 43, and 44) belonging to the assessment of children’s fundamental arithmetic skills (pre-mathematics skills) emerged as the easiest items, as evidenced by their remarkably low item discrimination scales, which ranged from 0.09 to 0.19. A discrimination value close to zero for these specific items suggests that they do not effectively differentiate between respondents of varying levels within the construct being measured by the DIFER test (Zwick et al. 1999). As a result, we opted to exclude these five items from the assessment of school readiness using the DIFER test to ensure its construct validity.

#### 4.1.1. Differential Item Functioning (DIF) for Age Groups

Subsequent to the elimination of the five least challenging question items from the dichotomous test, a differential item functioning (DIF) analysis of the DIFER test was performed using the Rasch model. This analysis aimed to probe how the test items operate in the context of distinct age groups, namely, the 4th, 5th, 6th, 7th, and 8th years. DIF evaluation can be approached from distinct methods; (1) through the consideration of statistically significant probability (*p* < 0.05), and (2) by examining the magnitudes of DIF. The classification of DIF magnitudes comprises three levels: minimal, slight to moderate (with/DIF/ ≥ 0.43 logits), and moderate to substantial (with/DIF/≥ 0.64 logits) (Zwick et al. 1999). The outcomes of this analysis indicated that the DIF logits significantly (* *p* < 0.05) fell within the range of 0.37 and −0.20 for the 4th-year age group; 0.29 and −0.12 for the 5th-year age group; 0.15 and −0.09 for the 6th-year age group; 0.09 and −1.23 for the 7th-year age group; and +0.18 and −0.29 for the 8th-year age group. It means that the DIFER test is significantly discriminative for different age groups, but negligible to change the items, recommended by Zwick et al. (1999). These findings potentially underlie the transformative impact of students’ age-related developmental shifts or their overarching general cognitive ability (g).

#### 4.1.2. Multidimensional Rasch Analysis

Moreover, we proceeded with a comprehensive multidimensional Rasch analysis to investigate the item-person parameters associated with the DIFER school readiness test. The validity of item and person fit was assessed using the root mean square (MNSQ) for infit/outfit measures, which fell within the recommended range of 0.5 to 1.15 as suggested by Andrich (2018). Since our sample consisted of more than 3000 students (Azizan et al. 2020), the z-standardized (ZSTD) infit/outfit measures for persons and items were not considered, as they tend to be less informative in larger samples where person abilities as latent traits can be differentiated. The item separation analysis indicated that all domains of the DIFER test contained a range of easy and difficult items, confirming its content validity (Boone et al. 2014). For this study, we evaluated each subtest (as unidimensional models) within the multidimensional model, following the recommendation by Bond and Fox (Bond and Fox 2015). The DIFER test was deemed suitable for assessing children’s school readiness based on an underlying construct consisting of distinct yet related dimensions. We also assessed unidimensionality and local independence. The raw variance by measure values for all tasks can be found in Table 4. The results demonstrated that the DIFER test achieved a satisfactory threshold of over 30% (Gliner et al. 2017). Moreover, the unexplained variance for the first contrast values was below 2 for all domains of the DIFER test, confirming unidimensionality and indicating that the test encompassed nearly all relevant dimensions based on the students’ readiness assessment. Local independence was supposed, signifying that each item in the DIFER test was independent. To determine local independence, we examined the raw residual correlation between item pairs. According to Boone et al. (2014), a raw residual correlation between item pairs below 0.3 is considered acceptable. Our results showed that the items from different domains of the DIFER test had residual correlations ranging from 0.09 to 0.29, which further supported the assumption of acceptable local independence.

### 4.2. Addressing RQ_2_

This research question aims to examine the reliability and validity of the DIFER test, a criterion-referenced test of school readiness in Hungary. We utilized IBM SPSS Statistics 23.0 to measure the reliabilities, means, standard deviations, and correlations. The Kaiser–Meyer–Olkin (KMO) test indicated the appropriateness of the data for the factor analysis. The DIFER test yielded a very good KMO value (KMO = 0.826). As per Gliner et al. (2017), a KMO value above 0.5 is acceptable, while a value above 0.7 is considered good. Hence, all dimensions of the school readiness DIFER test were deemed suitable for further analysis in assessing the reliability of the school readiness DIFER test.

**Figure 4:**
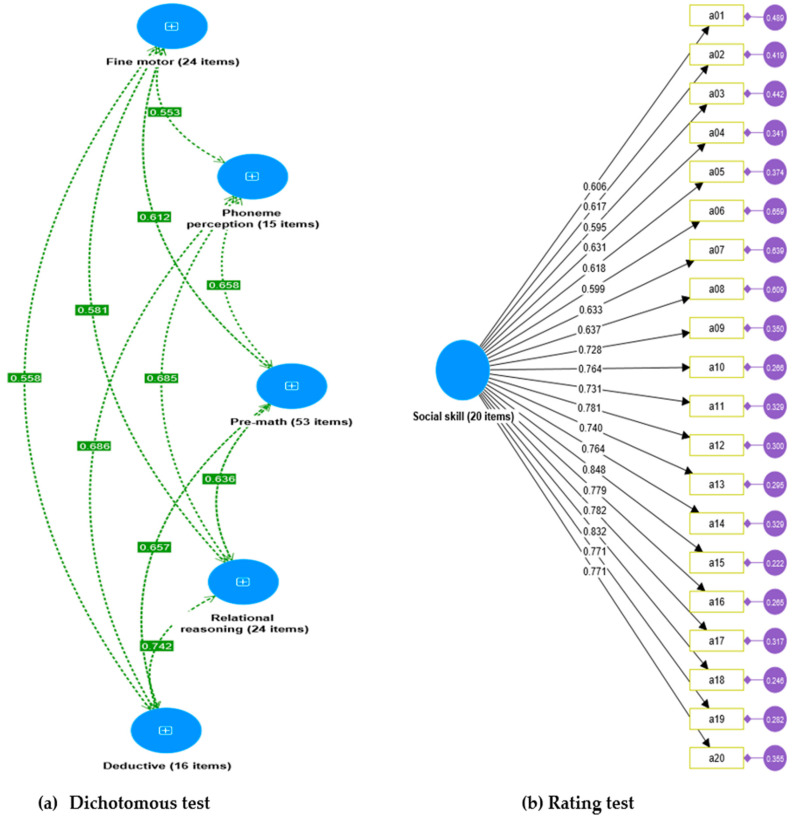
CFA model for five dimensions of DIFER (N = 3050).

Our findings revealed a good model fit, as indicated by non-significant chi-square (χ2) values and positive degrees of freedom (df), demonstrating the appropriateness of the DIFER test for assessing students’ school readiness. The fit indices, including the standardized root mean square residual (SRMR), comparative fit index (CFI), and root mean square error of approximation (RMSEA), were consistent with Kline’s (2015) recommendations and indicated a good model fit for the models (Table 5). Specifically, the SRMR provided a measure of the discrepancy between the observed and model-implied covariance matrices in the DIFER test. The CFI compared the fit of the hypothesized model to a baseline model, indicating how well the hypothesized model fit the observed data. The RMSEA described the amount of unexplained variance or error remaining after applying the model. In our CFA models as depicted in Figure 4, we examined the item-factor correlation coefficients ranging from 0.46 to 0.84. It is important to note that, despite including all the items from the dichotomous model in the analysis, they were omitted from the visual representation due to the large number of items (132 items) and to enhance the clarity of the unobserved domain variables. Our CFA models suggest the close relations between items and factors, supporting the development of strong constructs for both the dichotomous and rating versions of the DIFER school readiness assessment. Based on these results, we can conclude that the models are suitable for estimating the related measures of the school readiness assessment.

#### Correlational Changes among Factors for Different Age Groups

We have previously established the significant variations in DIF sizes or distinct evaluations across diverse age groups (4th, 5th, 6th, 7th, and 8th years), as presented earlier. Despite the observed substantial DIFER test differences among these age groups, we maintain the consistency of the factor structures across the age spectrum, primarily because the DIF sizes remained within the recommended parameters (/DIF/ ≤ 0.43).

Expanding on this, we extended our investigation to the correlations within the unaltered factor structures for the different age groups. This exploration aimed to quantify the range of differences in the correlations among the various factors within distinct age groups. For the 4th-year age group, the correlations spanned from low (r = 0.284) to moderate (r = 0.55) levels. Similarly, the 5th-year age group exhibited correlations ranging from low (r = 0.282) to moderate (0.512) levels. The 6th-year age group’s correlations ranged from low (r = 0.301) to moderate (r = 0.524) levels. The 7th-year age group showed correlations from low (r = 0.237) to moderate (r = 0.540) levels, while the 8th-year age group displayed correlations from low (r = 0.273) to moderate (r = 0.559) levels (Table 6).

These findings suggest that the presence of ‘g’ does not significantly vary among different age groups. It is reasonable to infer that the overall cognitive capability, commonly referred to as ‘g,’ exhibits minor fluctuations across different age groups in relation to their performance in the DIFER school readiness assessment tests. However, it is important to acknowledge that subtle variations in the ‘g’ effect among age groups might still exist, albeit not to a substantial degree.

Then, to ensure the construct validity of the DIFER, another examination was also conducted to determine if the behavior of the construct aligned with the theories mentioned earlier. Convergent validity and discriminant validity were assessed to establish the construct validity of the factors. Following the criteria proposed by Fornell and Larcker (1981) and Oo et al. (2023), factors within the same construct are considered valid if the average variance extracted (AVE) value exceeds 0.50, and their CR values exceeded 0.70, confirming convergent validity (Table 7). The evaluation of discriminant validity was constructed by employing the HTMT ratio as proposed by Henseler et al. (2015). The outcomes are presented in Table 8, demonstrating values spanning from 0.41 to 0.77. As all the values are below 0.85, the DIFER test demonstrated good discriminant validity. Based on the presented information regarding the reliability and validity assessments of the DIFER test, it can be inferred that the test is reliable and valid for measuring students’ school readiness.

### 4.3. Addressing RQ_3_

The third research question examines the measurement invariance of the school readiness DIFER test across different groups, including country, gender, and age of students. To establish a comparison standard for measurement invariance across these groups, a baseline model was initially constructed. Due to the use of two different tests in the DIFER assessment (dichotomous test and rating test), separate analyses of measurement invariance were conducted for each test.

Initially, the measurement invariance of the dichotomous test model was assessed within each group (country, gender, and age level), where no correlations among measurement errors were considered. However, the results of this analysis were unsatisfactory in terms of assessing the measurement invariance of the DIFER test (CFI = 0.760, RMSEA = 0.082, and SRMR = 0.092). Consequently, the next step involved analyzing the modification indices for each sample, allowing for correlations among measurement errors, as suggested by Kline (2015). The main objective at this stage was to identify a baseline model that would adequately fit all groups (country, gender, and age level) and establish measurement invariance. To achieve this, fit indices were calculated for the model with correlated errors within each sample for both the dichotomous test and the rating test of the DIFER. Correlations among measurement errors of specific items within the same factors were introduced for the dichotomous test (R6 and R7, R26 and R27, R27 and R28, and R43 and R44). Following the introduction of these correlations, the CFA model was re-evaluated, resulting in a good model fit for all dimensions of the dichotomous test. Similarly, for the rating test of DIFER, measurement errors of certain items were correlated (a04 and a05, a07 and a08, a15 and a18, and a16 and a19) to achieve a good fit for measuring variances across different groups. Consequently, a good model fit was attained for each group based on country, gender, and age levels, as indicated in Table 9.

#### 4.3.1. Measurement Invariance across Countries

The measurement invariance of the DIFER test across Slovakia and Hungary was examined through a series of analyses. Initially, the configural model was assessed, which demonstrated a strong baseline model fit for all indices in both the dichotomous and rating tests, as indicated in Table 10 and Table 11. Subsequently, metric invariance was evaluated by constraining the factor loadings to be equal across Hungarian students in both countries. Importantly, the comparison between the configural and metric models revealed no significant decrease in fit, indicating the full invariance of factor loadings across countries in both test formats (∆CFI = −0.001, −0.001, ∆RMSEA = −0.001, and ∆SRMR = −0.002). Further analysis focused on scalar invariance, where the intercepts of all items were constrained to be the same across the groups. Once again, the results demonstrated that the fit of the models did not significantly decrease in both the dichotomous and rating tests (∆CFI = −0.002, ∆RMSEA = 0.000, and ∆SRMR = −0.002). To assess residual invariance, item residuals were constrained in the partial scalar model. Encouragingly, the fit indices supported the adequacy of this residual model (∆CFI = −0.001, ∆RMSEA = 0., and ∆SRMR = −0.001), showing intercepts and residual variances exhibited partial invariance across countries. These findings align with the recommended thresholds for metric, scalar, and residual invariance (∆CFI < 0.01) (∆SRMR < 0.03) (∆RMSEA < 0.015) as outlined by Kline (2015). Accordingly, it indicates that the overall measurement invariance of the DIFER test between Slovakia and Hungary was upheld.

#### 4.3.2. Measurement Invariance across Genders

The adequacy of the configural model in representing the hypothesized relationships in the DIFER test for school readiness across gender was assessed. Both the dichotomous and rating tests of DIFER exhibited good model fits across all examined models, including configural, metric, scalar, and residual. The comparison between the configural and metric models met the predefined thresholds for fit indices (∆CFI = −0.001; ∆RMSEA = −0.001; and ∆SRMR = −0.002). There was no significant decrease in fit observed between the metric and scalar models (∆CFI = 0, ∆RMSEA = 0.001, and ∆SRMR = 0.001, −0.001). Furthermore, the fit indices of the residual invariance model were not significantly different from those of the scalar invariance model (∆CFI = −0.001, −0.002; ∆RMSEA = −0.001, −0.003; and ∆SRMR = −0.004, −0.002), as presented in Table 10 and Table 11. These findings suggest that the DIFER test maintains its measurement invariance across gender, supporting its reliability and validity in assessing school readiness.

#### 4.3.3. Measurement Invariance across Ages

The investigations into measurement invariance across different age groups (4th, 5th, 6th, 7th, and 8th) revealed that the configural, metric, and scalar models of both the dichotomous and rating tests demonstrated a good fit across all age groups (Table 10 and Table 11). However, when examining the full scalar or residual invariance of the dichotomous test, the fit indices indicated that the intercepts were not equal among the age groups (∆CFI = −0.020, ∆RMSEA = 0.017, and ∆SRMR = 0.022) (Table 10). To identify the specific item causing the misfit, we released the constraint on each intercept and found that item74, related to the pre-mathematics skills, was responsible for the change in CFI and RMSEA. By allowing this intercept to vary freely, there was no significant change in fit between metric and partial scalar models (∆CFI = −0.009, ∆RMSEA = 0.002, and ∆SRMR = 0.004). Therefore, we can conclude that there is partial invariance (all parameters are equal, but only item74 is variant) across the age groups of children in the DIFER assessment. These findings provide valuable insights into the measurement properties of the test (such as configural, metric, scalar, and residual) across different age groups.

#### 4.3.4. Latent Mean Differences

The intercepts of the observed variables of the DIFER test were equated across countries, genders, and ages, allowing for a meaningful comparison of latent means among young children. Notably, the measurement models presented in Table 8 and Table 9 displayed a satisfactory fit for scalar invariance across these factors, affirming the accuracy of the estimates obtained through this approach. Delving into the DIFER school readiness test, which encompassed six distant domains, intriguing findings emerged. Young students from Hungary exhibited a remarkable superiority in fine motor skills and social skills, surpassing their Slovakian counterparts by a significant margin (z = 7.173; z = 13.188). However, the tides shifted when it came to the remaining four skills—phoneme perception, pre-mathematics, relational reasoning, and deductive reasoning—where the latent abilities of Slovakian students surpassed those of their Hungarian peers. When dissecting the gender groups, a captivating distinction surfaced. Male students displayed a noteworthy advantage in fine motor skills (z = 9.462) and deductive reasoning skills (z = 10.943) compared to their female counterparts, highlighting their innate prowess in these areas (*p* < .001). However, no substantial disparities were detected in the remaining skills, indicating a relatively balanced distribution of latent abilities across genders (see Table 12).

Furthermore, an intriguing pattern emerged as we explored different age groups (4th, 5th, 6th, 7th, and 8th years) among young children. Evidently, a clear progression in latent abilities unfolded, with each higher age groups (6th, 7th, and 8th) demonstrating superior latent ability, e.g., in the pre-mathematics skills (z = 15.820), compared to the lower age groups (4th and 5th) (z = 8.097, *p* < .001). This compelling observation implies that, as children mature and advance in age, their latent abilities tend to flourish, culminating in a progressively enhanced skill set. Overall, these captivating insights shed light on the nuanced variations in latent abilities across countries, genders, and age groups, illuminating the diverse facets of young children’s developmental trajectories.

## 5. Discussion

To address the existing research gap regarding the evaluation of the psychometric properties of the DIFER assessment for Hungarian children in Slovakia and Hungary, this study aimed to investigate three specific research questions. By doing so, we planned to contribute to the understanding of the measurement qualities of the DIFER assessment and bridge the research gap in this area.

The first research question was to investigate the alignment between students’ abilities and the difficulty levels of items in the DIFER school readiness assessment. According to the item-response theory, it is also important to measure the relationship between items and students’ ability (Liu et al. 2022; Polat 2022). Therefore, to answer this question, we conducted a thorough analysis of the item-person parameters using Rasch analysis. This analysis enabled us to examine the relationship between students’ abilities and the difficulty levels of the test items, shedding light on the alignment between the two. The item-person maps presented in our findings provided a visual representation of this alignment, showing that students generally performed well on items of moderate difficulty. This observation suggests that the DIFER test effectively captures students’ abilities across a range of skill levels, allowing for a comprehensive assessment of school readiness. However, within the dichotomous test, we identified 5 items (out of 137 items) that emerged as particularly easy based on their low item discrimination scales. These items exhibited a limited ability to differentiate between students of varying ability levels within the construct being measured by the DIFER test. To ensure the construct validity of the assessment, we made a decision to exclude these items from further analyses. By doing so, we improved the sensitivity and accuracy of the DIFER test in assessing school readiness. This aligns with some studies that removed some items for their test accuracy (Veas et al. 2016; Yan and Mok 2012; Ziv 2013).

After removing the psychometric items, a comprehensive multidimensional Rasch analysis was conducted to examine the item-person parameters associated with the DIFER school readiness test. The validity of the item and person fit was evaluated using the recommended MNSQ for infit/outfit measures, which fell within the acceptable range. The satisfactory item separation analysis indicated that all domains of the DIFER test encompassed a range of items spanning different levels of difficulty, confirming the content validity of the assessment. The examination of unidimensionality and local independence also suggested to us that the DIFER test effectively assessed the relevant dimensions of school readiness (Soeharto and Csapó 2022). Hence, by addressing the first research question, we gained valuable insights into the difficulty levels of the DIFER assessment items, enabling us to make appropriate adjustments based on students’ ability levels for a more accurate and tailored school readiness assessment.

The second research question is to examine the reliability and validity of the DIFER school readiness assessment, using the perspective of the classical test theory as proposed by Haw et al. (2022). This examination of the psychometric properties of the DIFER test provides crucial insights into the assessment’s reliability and validity, which are fundamental aspects of any robust measurement tool. In order to evaluate the reliability of the DIFER test, several statistical measures were employed using IBM SPSS Statistics 23.0. Internal consistency, a commonly used indicator of reliability, was assessed through the estimation of Cronbach’s alpha and composite reliability (CR). The results indicated that the internal consistency reliability of all dimensions of the DIFER test exceeded the widely accepted threshold of 0.70. Additionally, the CR values for all dimensions surpassed the threshold of 0.70, further supporting the overall reliability of the DIFER test. This finding is also consistent with other school readiness assessments (Amukune et al. 2022a; Csapó et al. 2014; Józsa et al. 2022a), encompassing the internal consistency reliability of all dimensions of the DIFER test. In the reliability measure of the DIFER test, the high Cronbach’s alphas can potentially indicate item redundancies and narrow item construction. However, in the context of our DIFER test, we completely understand the significance of maintaining a balanced and diverse set of items that accurately assess the range of skills related to school readiness. Furthermore, the DIFER test is a criterion-referenced test in Hungary. Therefore, we could not delete many items. However, researchers in the future have the flexibility to adapt and verify the suitability of the DIFER school readiness test according to their particular circumstances.

To assess the construct validity of the DIFER test, a confirmatory factor analysis (CFA) was conducted using SmartPLS4. The results demonstrated a good fit between the hypothesized model and the observed data, as indicated by non-significant chi-square values, positive degrees of freedom, and favorable fit indices such as SRMR, CFI, and RMSEA. These fit indices, which align with Kline’s (2015) recommendations, provided evidence of a strong model fit for both the dichotomous and rating versions of the DIFER school readiness assessment. Further analysis of the CFA models revealed good item-factor correlation coefficients, indicating close relationships between the items and the underlying factors of both the dichotomous and rating tests. This finding supports the development of robust constructs for both tests by CFA measures (Diotaiuti et al. 2022; Liu et al. 2020). Consequently, it can be inferred that the DIFER test successfully captures the multidimensional nature of school readiness, lending further support to its construct validity. The construct validity of the DIFER test was further assessed through an examination of convergent validity and discriminant validity. The findings indicate that the DIFER test exhibits strong construct validity, aligning with established criteria for convergent and discriminant validity assessments (Russo et al. 2019).

In accordance with the theory of SEM, the third research question was aimed at investigating potential variations in performance on the DIFER test based on factors such as countries, genders, and ages. The measurement invariance of the DIFER test was examined across different groups, and separate analyses were conducted for the dichotomous test and rating test components. Initially, the measurement invariance of the dichotomous test model was assessed within each group, but the results indicated unsatisfactory fit indices. By addressing the measurement errors through the introduction of correlations, improvement was observed in the model fit for all dimensions of both the dichotomous and rating tests, aligning with findings from the previous studies (Calchei et al. 2023; Zewude and Hercz 2022). Measurement invariance was then examined across countries, genders, and the ages of 4th, 5th, 6th, 7th, and 8th years. The findings from the measurement invariance analyses provide valuable insights into the performance variations on the DIFER test based on country, gender, and age. The established measurement invariance across countries suggests that the test is valid and reliable for assessing school readiness (based on fine motor, phoneme perception, pre-mathematics, relational reasoning, deductive reasoning, and social skills) in both Slovakia and Hungary. Similarly, the measurement invariance across genders supports the use of the DIFER test as a fair assessment tool for both boys and girls. However, it is important to note that partial invariance was observed across age groups, specifically related to item74. This may be the reason that this item was somehow easy for assessing different age groups of students from both countries. Therefore, researchers from some studies (Kline 2015; Macy et al. 2022; Soeharto and Csapó 2022) suggested that huge number of participants and their different ages can also cause invariance in all types of assessments. This finding suggests that the interpretation of the test results should consider the potential influence of age on certain aspects of school readiness, particularly pre-mathematics skills.

Moreover, the results regarding latent mean differences in the DIFER test provide valuable insights into the variations observed across countries, genders, and age groups (Csapó et al. 2014; Józsa et al. 2017). In terms of country comparisons, Hungarian students who live in Hungary exhibited notable superiority in fine motor skills and social skills compared to those who live in Slovakia. When examining gender differences, male students demonstrated a significant advantage in fine motor skills and deductive reasoning compared to their female counterparts. However, no substantial disparities were found in the remaining skills. Exploring different age groups revealed a clear progression in latent abilities as children advanced in age (Anthony et al. 2022). Higher age groups (6th, 7th, and 8th years) exhibited superior latent abilities, particularly in areas such as pre-mathematics skills. Overall, these findings highlight the nuanced variations in latent abilities across countries, genders, and age groups, providing valuable insights into the diverse developmental trajectories of young children. It underscores the importance of considering multiple factors (fine motor skills, phoneme perception, pre-mathematics skills, relational reasoning, deductive reasoning, and social skills) when assessing school readiness and emphasizes the need for tailored educational approaches that accommodate individual strengths and developmental trajectories (Józsa et al. 2022a).

In the DIF analysis, our exploration into how the test items functioned across distinct age groups shows intriguing disparities. Notably, the DIF logits exhibited a significant range between the 4th-year and 8th-year age groups. These observations underscore that the cognitive demands of certain items are influenced by age, implying an intricate interplay between cognitive maturation and item performance. This insight aligns with the prevailing theoretical considerations regarding the developmental trajectory of general cognitive ability (g) and its potential evolution across childhood (Demetriou et al. 2020; Neumann et al. 2021). However, the MI analysis, which explored the equivalence of the measurement properties across the same age groups, presents a contrasting yet equally significant dimension. The robustness of our measurement model across various age groups is evident through the consistent fit of the configural, metric, and scalar models for both the dichotomous and rating tests. The reconciliation of these two results can be framed within the context of the developmental dynamics of the ‘g’ factor. The DIF findings potentially reflect the evolving cognitive capabilities of children as they progress through different age groups, mirroring the theoretical anticipation of cognitive differentiation with age (Demetriou et al. 2020). On the other hand, the MI results indicate that while the overall measurement structure remains stable across ages, specific item behaviors may undergo slight variations. This interplay could be indictive of age-related cognitive shifts impacting the understanding and mastery of certain skills, such as pre-mathematics abilities. Further research is warranted to delve deeper into the nature of these age-related cognitive dynamics, considering the intricate interplay of ‘g’ and domain-specific cognitive abilities across developmental stages.

The study has some limitations. This study focuses on assessing school readiness during the DIFER test, but does not include other potential external factors that may influence readiness such as socioeconomic status, parental involvement, or early childhood education experiences. The findings of the research were interpreted within the context of the DIFER test and the population studied, and thus, applying the results to other populations should be considered in future research.

## 6. Conclusions

In conclusion, this study was conducted to investigate the psychometric properties of the DIFER school readiness assessment. The findings provide important insights into the alignment of students’ abilities with the item levels in the DIFER test, the reliability and validity of the test, and the invariance in test performance based on countries, genders, and ages. The DIFER test effectively measured the intended constructs of school readiness, ensuring that the test items appropriately correspond to students’ abilities. Moreover, the tests showed satisfactory levels of convergent validity and discriminant validity, as well as high values for AVE and CR, suggesting the suitability of the DIFER test for assessing school readiness. Moreover, the analysis of measurement invariance across countries, genders, and age groups revealed a lack of significant variance in the DIFER school readiness assessment, with the exception of a few differences in latent means.

Based on these findings, it is suggested that we further explore the factors that contribute to the observed latent mean differences in the DIFER school readiness assessment across countries, genders, and age groups. Additionally, conducting qualitative research or employing additional measures could provide deeper insights into the underlying reasons behind these variations. Further investigations into the contextual and cultural factors that may influence children’s development and performance on the DIFER test could also be beneficial. This additional research can contribute to a more comprehensive understanding of the complexities involved in assessing school readiness and inform targeted interventions and support for children in different ways. The findings of this research contribute to our understanding of the complexity of the school readiness assessment and provide valuable insights for educational practitioners and policymakers in supporting children’s developmental needs in such skills as fine motor, phoneme perception, pre-mathematics, relational reasoning, deductive reasoning, and social skills. The educators can utilize the DIFER test as a robust and valid tool for assessing children’s school readiness. Furthermore, this study contributes to the growing body of literature on psychometric assessment in education, providing valuable guidance for practitioners seeking reliable and valid tools to assess children’s readiness for formal education.

## Figures and Tables

**Table 1:** Characteristics of children’s school readiness assessments in Hungary.

Instruments	Authors (Time)	Contents/Factors	Assessor	Students	Reliability	MI	Study	Country
PREFER	Nagy (1976)	✓ Mother tongue ✓ Mathematics ✓ Manipulative thinking ✓ Writing motor co-ordination ✓ Self-help ✓ Task relation ✓ Attitude	Teachers/ examiners	Children aged 5–6 years	-	-	National survey	Hungary
DIFER	Nagy et al. (2004a)	✓ Pre-mathematics, ✓ Fine motor control ✓ Phoneme perception ✓ Comprehension of cause and effect ✓ Deductive reasoning ✓ Vocabulary related to relationships ✓ Social skills	Teachers/ examiners	Children aged 4–8 years	Standardized as national test	-	National survey	Hungary
DIFER	Józsa and Fenyvesi (2006)	✓ Writing–movement co-ordination ✓ Speech-language listening ✓ Relational vocabulary ✓ Elementary arithmetic ✓ Empirical reasoning ✓ Empirical contextual understanding	Teachers	Students with learning disabilities, aged 7–8	-	-	Simple survey	Hungary
Computer-based DIFER	Csapó et al. (2014)	✓ Speech sound discrimination ✓ Relational reasoning ✓ Counting/basic numeracy ✓ Deductive reasoning ✓ Inductive reasoning	Teachers	First-grade students	Cronbach’s alpha	MG-CFA for media effects	Simple survey	Hungary
A game-like, computer-based assessment	Józsa et al. (2017)	✓ Mastery motivation ✓ Executive functions ✓ Pre-academic skills	Trained examiners	Students aged 3–8 years	-	-	Cross-cultural	Hungary and America
DIFER	Józsa and Barrett (2018)	✓ Social skills	Trained examiners	Children aged around 5 years	Cronbach’s alpha	-	Longitudinal study	Hungary
DIFER	Józsa et al. (2022b)	✓ Pre-maths ✓ Phoneme perception ✓ Vocabulary of relations ✓ Social skills ✓ Fine motor skills	Trained examiners	Preschool children	Cronbach’s alpha	-	Longitudinal (8 years)	Hungary
FOCUS app (a game-like tablet-based assessment)	Józsa et al. (2022a)	✓ Mastery motivation ✓ Executive functions ✓ Pre-academic skills	Trained examiners	Students aged 3–8 years	-	-	Cross-cultural	Hungary and Kenya
CHEXI	Amukune et al. (2022b)	✓ Working memory ✓ Inhibition ✓ Regulation ✓ Planning	Teachers	Preschool children	Cronbach’s alpha	MG-CFA	Cross-cultural	Hungary and Kenya
Computer-based DIFER	Molnár and Hermann (2023)	✓ Counting and basic numeracy skills ✓ Pre-cursors of reading skills ✓ Inductive reasoning	Trained examiners	First-grade students	EAP reliability	-	Longitudinal study (before/after COVID)	Hungary

**Table 2:** Number of participants for each country divided by gender and age groups.

Variable	Slovakia	Hungary	Total
Number of Participants	1609 (52.75%)	1441 (47.25%)	3050
***Gender***			
Male	779 (47.5%)	862 (52.5%)	1641
Female	830 (58.87%)	579 (41.13%)	1409
***Age***			
4th year	159 (56.38%)	123 (43.62%)	282
5th year	370 (56.74%)	282 (43.26%)	652
6th year	429 (51.56%)	403 (48.44%)	832
7th year	351 (50.87%)	339 (49.13%)	690
8th year	300 (50.51%)	294 (49.49%)	594

**Table 3:** Preliminary analyses for the school readiness assessment.

DIFER	Fine Motor	Phoneme Perception	Pre-Maths	Relational Reasoning	Deductive Reasoning	Social Skills	Total
N of items	24	15	58	24	16	20	157
Mean	13.08	12.4	40.55	19.54	10.63	81.16	71.97
SD	6.6	2.59	12.7	3.86	4.12	12.77	16.04
Skewness	−0.04	−1.18	−0.64	−1.12	−0.77	−0.723	0.64
Kurtosis	−0.949	1.36	−0.43	1.7	−0.05	0.51	−0.02

**Table 4:** Summary for the Rasch parameters for the school readiness test, DIFER.

Psychometric Properties	Fine Motor Skills	Phoneme Perception	Pre-Maths	Relational Reasoning	Deductive Reasoning	Social Skills
N of items	24	15	53	24	16	20
Mean	0.29	2.16	1.74	1.54	79	2.14
SD	1.94	1.43	2.66	0.98	1.29	1.59
MNSQ (item-infit)	0.99	1	0.98	1.00	1.01	0.99
MNSQ (item-outfit)	1.11	0.97	1.99	1.00	0.98	1.01
MNSQ (person-infit)	0.99	1.00	0.97	1.00	1.00	1.01
MNSQ (person-outfit)	1.04	0.97	1.2	1.00	0.98	1.01
Item separation	32.33	10.11	35.90	11.05	14.80	14.78
Person separation	2.79	2.72	4.26	3.44	2.65	3.07
***Unidimensionality***						
Raw variance by measure	34.50%	38.2%	38.3%	38%	40.36%	61.26%
Unexplained variance 1**st** contrast	1.45	1.42	1.13	1.62	1.84	1.32

**Table 5:** Model fit measures for the DIFER assessment.

DIFER	Items	Chisqr/df	*p* Value	Absolute Index, SRMR (<0.08 *)	Comparative Index, CFI (>0.90 *)	Parsimonious Index, RMSEA (<0.06 *)
Dichotomous test	132	2.85	0.052	0.08	0.90	0.057
Rating test	20	2.50	0.073	0.07	0.92	0.046

Note. * shows the recommended values.

**Table 6:** Factor correlations for different age groups.

Age 4	2	3	4	5	6
1. Social skills	0.284 **	0.446 **	0.374 **	0.432 **	0.452 **
2. Fine motor skills		0.256 **	0.282 **	0.306 **	0.357 **
3. Phoneme perception			0.577 **	0.504 **	0.526 **
4. Relational reasoning				0.489 **	0.486 **
5. Deductive reasoning					0.529 **
6. Pre-maths skills					
**Age 5**	2	3	4	5	6
1. Social skills	0.282 **	0.381 **	0.367 **	0.413 **	0.465 **
2. Fine motor skills		0.324 **	0.305 **	0.292 **	0.429 **
3. Phoneme perception			0.512 **	0.479 **	0.473 **
4. Relational reasoning				0.500 **	0.510 **
5. Deductive reasoning					0.485 **
6. Pre-maths skills					
**Age 6**	2	3	4	5	6
1. Social skills	0.301 **	0.351 **	0.413 **	0.425 **	0.334 **
2. Fine motor skills		0.334 **	0.279 **	0.335 **	0.430 **
3. Phoneme perception			0.465 **	0.462 **	0.504 **
4. Relational reasoning				0.515 **	0.524 **
5. Deductive reasoning					0.464 **
6. Pre-maths skills					
**Age 7**	2	3	4	5	6
1. Social skills	0.237 **	0.414 **	0.373 **	0.417 **	0.358 **
2. Fine motor skills		0.274 **	0.318 **	0.314 **	0.457 **
3. Phoneme perception			0.487 **	0.447 **	0.485 **
4. Relational reasoning				0.540 **	0.463 **
5. Deductive reasoning					0.453 **
6. Pre-maths skills					
**Age 8**	2	3	4	5	6
1. Social skills	0.273 **	0.373 **	0.349 **	0.393 **	0.440 **
2. Fine motor skills		0.289 **	0.274 **	0.264 **	0.330 **
3. Phoneme perception			0.559 **	0.533 **	0.441 **
4. Relational reasoning				0.505 **	0.543 **
5. Deductive reasoning					0.434 **
6. Pre-maths skills					

Note: ** *p* < .01.

**Table 7:** Convergent validity of DIFER.

Dimensions	N of Items	Mean (SD)	Cronbach’s Alpha	CR	AVE
			(>0.60) *	(>0.70) *	(>0.50) *
Fine motor skills	24	13.08 (6.60)	0.92	0.72	0.50
Phoneme perception	15	12.40 (2.59)	0.74	0.92	0.63
Pre-mathematics	53	40.55 (12.70)	0.95	0.96	0.65
Relational reasoning	24	19.54 (3.86)	0.80	0.86	0.55
Deductive reasoning	15	10.68 (4.12)	0.86	0.71	0.50
Social skills	20	81.16 (12.77)	0.95	0.94	0.51
Total	152	71.97(16.04)	0.97	0.86	0.55

Note: (*) recommended values.

**Table 8:** HTMT ratio for the discriminant validity of DIFER.

Construct	1	2	3	4	5	6
1. Fine motor skills		0.69	0.76	0.68	0.73	0.41
2. Phoneme perception			0.74	0.66	0.77	0.54
3. Relational reasoning				0.74	0.72	0.48
4. Deductive reasoning					0.69	0.47
5. Pre-mathematics						0.50
6. Social skills						

Note: HTMT (heterotrait–monotrait) ratio = average heterotrait–heteromethod correlations/square root of (average monotrait–heteromethod correlation of (first construct) × (second construct)).

**Table 9:** Fit indices of baseline model for each group of country, gender, and age levels.

DIFER	Groups	χ2 (df)	CFI	RMSEA [90% CI]	SRMR
Dichotomous test	Slovakia	145,555.9 (17,005)	0.942	0.050 [0.050, 0.050]	0.060
	Hungary	145,586.9 (17,005)	0.943	0.050 [0.050, 0.050]	0.060
	Male	117,642.8 (8778)	0.948	0.051 [0.049, 0.052]	0.060
	Female	114,522.7 (8778)	0.949	0.050 [0.049, 0.052]	0.059
	4th year	117,882.8 (17,002)	0.912	0.060 [0.059, 0.062]	0.063
	5th year	117,892.7 (17,002)	0.912	0.060 [0.059, 0.062]	0.063
	6th year	118,222.8 (17,002)	0.911	0.058 [0.058, 0.058]	0.060
	7th year	117,892.7 (17,002)	0.932	0.058 [0.058, 0.058]	0.061
	8th year	118,222.8 (17,002)	0.921	0.057 [0.055, 0.060]	0.065
Rating test	Slovakia	72,774.0 (210)	0.931	0.065 [0.063, 0.066]	0.063
	Hungary	69,876.9 (210)	0.931	0.065 [0.065, 0.065]	0.061
	Male	7051.8 (210)	0.939	0.063 [0.060, 0.065]	0.062
	Female	7308.7 (210)	0.940	0.060 [0.058, 0.062]	0.060
	4th year	3907.1 (210)	0.943	0.039 [0.059, 0.062]	0.034
	5th year	3831.8 (210)	0.947	0.043 [0.041, 0.044]	0.033
	6th year	3994.1 (210)	0.965	0.038 [0.037, 0.040]	0.044
	7th year	4045.6 (239)	0.914	0.047 [0.046, 0.048]	0.049
	8th year	5515.2 (265)	0.922	0.039 [0.038, 0.040]	0.042

**Table 10:** Testing measurement invariance of DIFER (dichotomous test) across country, gender, and age.

Models	χ^2^ (df)	CFI	RMSEA [90% CI]	SRMR	∆CFI	∆RMSEA	∆SRMR	MI
**MI across country (*N_Slovakia_ = 1609; N_Hungary_ = 1441*)**								
Configural	145,587.9 (17,008)	0.942	0.050 [0.050, 0.050]	0.060	-	-	-	-
Metric	146,010.3 (17,135)	0.941	0.050 [0.046, 0.050]	0.060	−0.001	0.000	0.000	Yes
Scalar	146,640.7 (17,267)	0.939	0.050 [0.046, 0.050]	0.057	−0.002	0.000	−0.003	Yes
Residual	146,653.8 (17,282)	0.938	0.050 [0.046, 0.050]	0.058	−0.001	0.000	0.001	Yes
**MI across gender (*N_male_ = 1641; N_female_ = 1409*)**								
Configural	117,642.8 (8778)	0.947	0.049 [0.049, 0.049]	0.056	-	-	-	-
Metric	116,114.5 (17,402)	0.947	0.049 [0.049, 0.049]	0.056	0.000	0.000	0.000	Yes
Scalar	146,114.5 (17,402)	0.947	0.049 [0.049, 0.049]	0.057	0.000	0.001	0.001	Yes
Residual	146,122.4 (17,408)	0.946	0.047 [0.045, 0.048]	0.053	−0.001	−0.001	−0.004	Yes
**MI across age (*N_year4_ = 282; N_yeat5_ = 652; N_yeat6_ = 832; N_yeat7_ = 690; N_yeat8_ = 594*)**								
Configural	116,845.9 (17,477)	0.921	0.059 [0.057, 0.060]	0.056	−	−	−	−
Metric	116,779.5 (17,489)	0.920	0.059 [0.055, 0.059]	0.056	−0.001	0.000	0.000	Yes
Scalar	146,884.5 (17,405)	0.920	0.050 [0.049, 0.050]	0.057	0.000	0.009	0.001	Yes
Residual	146,799.4 (17,411)	0.900	0.067 [0.077, 0.078]	0.079	−0.020	0.017	0.022	No
Residual (item74)	146,712.8 (17,400)	0.912	0.048 [0.046, 0.050]	0.055	−0.008	−0.002	−0.008	Yes

**Table 11:** Testing measurement invariance of DIFER (rating test) assessment across country, gender, and age.

Models	χ2 (df)	CFI	RMSEA [90% CI]	SRMR	∆CFI	∆RMSEA	∆SRMR	MI
**MI across country (*N_Slovakia_ = 1609; N_Hungary_ = 1441*)**								
**Configural**	4090.4 (298)	0.930	0.063 [0.050, 0.050]	0.062	−	−	−	−
**Metric**	4130.7 (317)	0.929	0.062 [0.061, 0.065]	0.060	−0.001	−0.001	−0.002	Yes
**Scalar**	4247.5 (337)	0.929	0.062 [0.060, 0.063]	0.060	0.000	0.000	0.000	Yes
**Residual**	4248.6 (332)	0.929	0.062 [0.060, 0.063]	0.060	0.000	0.000	0.000	Yes
**MI across gender (*N_male_ = 1641; N_female_ = 1409*)**								
**Configural**	3550.5 (298)	0.939	0.058 [0.049, 0.052]	0.06	−	−	−	−
**Metric**	3574.5 (317)	0.938	0.057 [0.056, 0.060]	0.056	−0.001	−0.001	−0.004	Yes
**Scalar**	3653.3 (337)	0.938	0.057 [0.055, 0.058]	0.055	0.000	0.000	−0.001	Yes
**Residual**	3661.5 (338)	0.936	0.054 [0.053, 0.056]	0.053	−0.002	−0.003	−0.002	Yes
**MI across age (*N_year4_ = 282; N_yeat5_ = 652; N_yeat6_ = 832; N_yeat7_ = 690; N_yeat8_ = 594*)**								
**Configural**	5533.8 (1007)	0.912	0.038 [0.037, 0.039]	0.035	−	−	−	−
**Metric**	5654.8 (1027)	0.910	0.038 [0.037, 0.039]	0.035	−0.002	0.000	0.000	Yes
**Scalar**	5654.8 (1028)	0.910	0.038 [0.037, 0.039]	0.034	0.000	0.000	0.001	Yes
**Residual**	5792.6 (1069)	0.908	0.038 [0.037, 0.039]	0.034	−0.002	0.000	0.000	Yes

**Table 12:** Comparison of latent mean differences on DIFER scales.

Group	DIFER Scales	Estimate	SE	CR Score	*p* Value
Country (Slovakia vs. Hungary)	✓ Fine motor skills	0.004	0.001	6.166 (7.173)	<.001
	✓ Phoneme perception	0.007	0.001	5.308 (4.968)	<.001
	✓ Pre-mathematics	0.004	0.001	7.466 (7.007)	<.001
	✓ Relational reasoning	0.002	0.000	3.226 (2.918)	<.01
	✓ Deductive reasoning	0.047	0.005	10.047 (9.629)	<.001
	✓ Social skills	0.251	0.021	12.024 (13.188)	<.001
Gender (Male vs. Female)	✓ Fine motor skills	0.006	0.001	9.462 (8.233)	<.001
	✓ Phoneme perception	0.007	0.001	7.264 (8.454)	<.001
	✓ Pre-mathematics	0.005	0.000	10.331 (11.45)	<.001
	✓ Relational reasoning	0.001	0.000	4.364 (4.671)	<.001
	✓ Deductive reasoning	0.046	0.003	10.943 (9.842)	<.001
	✓ Social skills	0.295	0.023	12.896 (12.040)	<.001
4th year vs. 5th year	✓ Fine motor skills	0.006	0.001	9.462 (9.233)	<.001
	✓ Phoneme perception	0.007	0.001	7.264 (8.454)	<.001
	✓ Pre-mathematics	0.260	0.032	8.097 (8.079)	<.001
	✓ Relational reasoning	0.001	0.000	4.364 (4.671)	<.01
	✓ Deductive reasoning	0.046	0.003	10.943 (9.842)	<.001
	✓ Social skills	0.282	0.018	15.820 (15.820)	<.001
4th year vs. 6th year	✓ Fine motor skills	0.006	0.001	9.462 (10.243)	<.001
	✓ Phoneme perception	0.007	0.001	7.264 (7.474)	<.001
	✓ Pre-mathematics	0.260	0.032	8.097 (9.179)	<.001
	✓ Relational reasoning	0.001	0.000	4.364 (5.672)	<.001
	✓ Deductive reasoning	0.046	0.003	10.943 (11.892)	<.001
	✓ Social skills	0.282	0.018	15.820 (15.820)	<.001
4th year vs. 7th year	✓ Fine motor skills	0.006	0.001	9.462 (9.244)	<.001
	✓ Phoneme perception	0.007	0.001	7.264 (11.459)	<.001
	✓ Pre-mathematics	0.260	0.032	8.097 (9.079)	<.001
	✓ Relational reasoning	0.001	0.000	4.364 (5.671)	<.01
	✓ Deductive reasoning	0.046	0.003	10.943 (11.842)	<.001
	✓ Social skills	0.282	0.018	15.820 (15.820)	<.001
4th year vs. 8th year	✓ Fine motor skills	0.006	0.001	9.462 (11.256)	<.001
	✓ Phoneme perception	0.007	0.001	7.264 (9.334)	<.001
	✓ Pre-mathematics	0.260	0.032	8.097 (8.979)	<.001
	✓ Relational reasoning	0.001	0.000	4.64 (4.699)	<.001
	✓ Deductive reasoning	0.046	0.003	10.943 (9.842)	<.05
	✓ Social skills	0.282	0.018	15.820 (15.820)	<.001
5th year vs. 6th year	✓ Fine motor skills	0.349	0.021	16.820 (16.999)	<.001
	✓ Phoneme perception	0.288	0.017	17.425 (18.898)	<.001
	✓ Pre-mathematics	0.270	0.017	15.447 (11.453)	<.001
	✓ Relational reasoning	0.312	0.020	15.677 (14.679)	<.001
	✓ Deductive reasoning	0.279	0.016	17.029 (19.842)	<.001
	✓ Social skills	0.295	0.023	12.896 (12.870)	<.001
5th year vs. 7th year	✓ Fine motor skills	0.349	0.021	16.820 (18.779)	<.001
	✓ Phoneme perception	0.288	0.017	17.425 (18.890)	<.001
	✓ Pre-mathematics	0.270	0.017	15.447 (15.665)	<.001
	✓ Relational reasoning	0.312	0.020	15.677 (18.556)	<.01
	✓ Deductive reasoning	0.279	0.016	17.029 (19.842)	<.001
	✓ Social skills	0.295	0.023	12.896 (12.870)	<.001
5th year vs. 8th year	✓ Fine motor skills	0.349	0.021	16.820 (17.001)	<.001
	✓ Phoneme perception	0.288	0.017	17.425 (20.448)	<.001
	✓ Pre-mathematics	0.270	0.017	15.447 (19.677)	<.001
	✓ Relational reasoning	0.312	0.020	15.677 (18.679)	<.01
	✓ Deductive reasoning	0.279	0.016	17.029 (19.842)	<.001
	✓ Social skills	0.295	0.023	12.896 (12.870)	<.001
6th year vs. 7th year	✓ Fine motor skills	0.006	0.001	9.462 (8.233)	<.001
	✓ Phoneme perception	0.007	0.001	7.264 (8.454)	<.001
	✓ Pre-mathematics	0.282	0.018	15.820 (15.820)	<.001
	✓ Relational reasoning	0.001	0.000	4.364 (4.671)	<.001
	✓ Deductive reasoning	0.046	0.003	10.943 (9.842)	<.001
	✓ Social skills	0.295	0.023	12.896 (12.040)	<.001
6th year vs. 8th year	✓ Fine motor skills	0.006	0.001	9.462 (8.233)	<.001
	✓ Phoneme perception	0.007	0.001	7.264 (8.454)	<.001
	✓ Pre-mathematics	0.282	0.018	15.820 (15.820)	<.001
	✓ Relational reasoning	0.001	0.000	4.364 (4.671)	<.01
	✓ Deductive reasoning	0.046	0.003	10.943 (9.842)	<.001
	✓ Social skills	0.295	0.023	12.896 (12.040)	<.001
7th year vs. 8th year	✓ Fine motor skills	0.373	0.011	32.905 (8.233)	<.001
	✓ Phoneme perception	0.302	0.009	33.452 (8.454)	<.001
	✓ Pre-mathematics	0.282	0.018	15.820 (15.820)	<.001
	✓ Relational reasoning	0.282	0.003	32.746 (4.671)	<.01
	✓ Deductive reasoning	0.252	0.008	31.015 (9.842)	<.05
	✓ Social skills	0.295	0.023	12.896 (12.040)	<.001

## Data Availability

Data are unavailable due to privacy or ethical restrictions.
